# Ambient air pollution and survival among Black women with epithelial ovarian cancer across diverse geographical regions of the United States

**DOI:** 10.1097/EE9.0000000000000426

**Published:** 2025-10-17

**Authors:** Ekaterina Chirikova, Courtney E. Johnson, Anke Huels, Pushkar P. Inamdar, Elisa V. Bandera, Lawrence H. Kushi, Jennifer A. Doherty, Joellen M. Schildkraut, Hari S. Iyer, Melissa Bondy, Edward S. Peters, Kendra Ratnapradipa, Jeffrey Marks, Christopher Pierson, Theresa Hastert, Kristin Haller, Grace Christensen, Salma Shariff-Marco, Scarlett L. Gomez, Andrew Lawson

**Affiliations:** a Department of Epidemiology & Biostatistics, University of California, San Francisco, California; b Department of Epidemiology, Emory University Rollins School of Public Health, Atlanta, Georgia; c Gangarosa Department of Environmental Health, Emory University Rollins School of Public Health, Atlanta, Georgia; d Department of Biostatistics and Bioinformatics, Emory University Rollins School of Public Health, Atlanta, Georgia; e Cancer Epidemiology and Health Outcomes, Rutgers Cancer Institute, New Brunswick, New Jersey; f Division of Research, Kaiser Permanente Northern California, Pleasanton, California; g Department of Population Health Sciences, Huntsman Cancer Institute, University of Utah, Salt Lake City, Utah; h Department of Epidemiology and Population Health, Stanford University School of Medicine, Stanford University, Stanford, California; i Department of Epidemiology, College of Public Health, University of Nebraska Medical Center, Omaha, Nebraska; j Department of Surgery, School of Medicine, Duke University, Durham, North Carolina; k Department of Oncology, School of Medicine, Wayne State University, Detroit, Michigan; l Helen Diller Family Comprehensive Cancer Center, University of California, San Francisco, California; m Department of Public Health Sciences, College of Medicine, Medical University of South Carolina, Charleston, South Carolina; n Usher Institute, School of Medicine, University of Edinburgh, Edinburgh, United Kingdom

## Abstract

**Background:**

Ovarian cancer is a leading cause of gynecologic cancer mortality, with Black women experiencing 5-year survival rates of only 41%. Disproportionate air pollution exposure may impact survival. We evaluated associations of fine particulate matter (PM_2.5_) and nitrogen dioxide (NO_2_) exposure with survival among Black women with epithelial ovarian cancer using data from the California Cancer Registry (CCR, n = 540) and the multi-state African American Cancer Epidemiology Study (AACES, n = 766).

**Methods:**

Annual PM_2.5_ and NO_2_ levels were estimated at a 1 km resolution using well-validated ensemble-based prediction models derived from the Socioeconomic Data and Application Center and assigned to the participants’ residential addresses per their year of diagnosis (2004−2016). Weibull accelerated failure time models with participant-level frailty were used to assess air pollutant exposure associations with overall survival.

**Results:**

Average PM_2.5_ and NO_2_ exposures were 11.3 μg/m³ and 25.8 ppb in CCR and 9.7 μg/m³ and 17.5 ppb in AACES. There was little evidence of an association between air pollution exposures and survival, with event time ratios (> 1 indicate longer survival) in CCR of 1.08 (95% CI = 0.97, 1.20) per 1 μg/m³ PM_2.5_ and 1.07 (95% CI = 0.99, 1.15) per 10 ppb NO_2_, and in AACES of 1.00 (95% CI = 0.93, 1.07) per 1 μg/m³ PM_2.5_ and 1.04 (95% CI = 0.91, 1.19) per 10 ppb NO_2_.

**Conclusions:**

Findings were modest and consistent across both cohorts and sensitivity analyses, supported by the use of advanced exposure modeling. Future research should use time-varying, long-term exposure data and examine interactions with occupation, physical activity, and neighborhood stressors.

**What this study adds**

- This is the first multi-state study to examine air pollution and survival in Black women with ovarian cancer using advanced exposure modeling. We found little evidence that PM_2.5_ or NO_2_ exposures impact survival, with consistent results across two diverse cohorts, sensitivity analyses, and comparisons with prior positive studies. Given the stark survival disadvantage for Black women with ovarian cancer (41% vs. 51% across all races), this study examines air pollution as a potential contributor to their poor survival. While findings suggest a limited role, they highlight the need to further improve exposure measurement and investigate other contextual factors.

## Introduction

Ovarian cancer is the sixth leading cause of cancer death among women in the United States, with 20,890 new cases and 12,730 deaths estimated for 2025.^1^ The 5-year survival for ovarian cancer in the United States is only 51%.^1^ However, substantial heterogeneity in ovarian cancer outcomes is observed across racial and ethnic groups, with Black women experiencing the lowest 5-year survival (41% in 2023).^2^ Black women are also less likely to receive guideline-recommended treatment for epithelial ovarian cancer (EOC),^3–6^ which accounts for more than 90% of all ovarian cancers.^7^ Established prognostic factors, including clinical and tumor characteristics, as well as insurance and socioeconomic status, do not appear to completely account for the 29% increased risk of death from EOC among Black women in the United States.^3^

The reasons for the observed differences in survival time among racial and ethnic groups are not well understood. While there is increasing evidence of the relevance of different domains of structural, environmental, and neighborhood context in relation to cancer survival disparities,^8–11^ the research on the impact of these factors on disparities in ovarian cancer is limited. To date, only a few studies have assessed the association of neighborhood factors with ovarian cancer outcomes, focusing on aspects such as residential segregation, area deprivation index, state and county of residence, rurality, travel time to healthcare facilities, and environmental exposures.^12^

Air pollution is a well-documented risk factor for cardiovascular disease (CVD) and lung cancer, as well as for CVD-related mortality among cancer survivors.^13^ Epidemiologic evidence on the detrimental effects of air pollution exposure on ovarian cancer incidence or mortality is sparse and remains inconclusive. Interest in this topic is driven by established links between ovarian cancer and various environmental and occupational exposures, including chemicals such as talc, pesticides, herbicides, and diesel or gasoline engine exhaust.^14,15^ With regard to ambient air pollution specifically, a large prospective cohort study found no statistically significant association between ovarian cancer incidence and time-varying fine particulate matter (PM_2.5_) exposure but reported a significant relationship with time-varying nitrogen dioxide (NO_2_) exposure.^16^ Several studies have reported a positive association of exposure to NO_2_ and PM_2.5_ with ovarian cancer mortality or survival,^17–20^ while others have found no significant relationship.^21,22^ Furthermore, disparities in air pollution exposure have been observed across geographic regions and among populations that differ by socioeconomic status, race, and ethnicity.^13^ This raises the question of whether poor survival rates observed among Black women may, in part, be attributed to disproportionate air pollution exposure—a relationship that remains underexamined in this population.

Our study addresses this evidence gap by exploring how long-term exposure to ambient air pollution (PM_2.5_ and NO_2_) influences EOC survival among Black women across different regions in the United States, leveraging data from the California Cancer Registry (CCR), the multi-state African American Cancer Epidemiology Study (AACES), and state-of-the-art high-resolution air pollution estimates.

## Materials and methods

### Study population

The study population was derived from two distinct sources: AACES^23,24^ and CCR,^25^ with analyses conducted separately for each cohort.

#### African American Cancer Epidemiology Study

AACES is a cohort of self-identified Black or African American women who were diagnosed with EOC, which has been described in detail elsewhere.^23,24^ Informed consent was obtained from all individual participants included in the study. The Institutional Review Board (IRB) at the Duke University Medical Center, the University of Virginia, and the IRBs of participating institutions, including the state and Surveillance, Epidemiology, and End Results (SEER) registries, approved the study protocol, consent documents, and questionnaires.

For this analysis, we leveraged data from Phase 1 of the AACES study, which enrolled women diagnosed with EOC between 2010 and 2015 via rapid case ascertainment and included 11 US sites that were selected based on having large African American populations across a variety of geographical areas. These included Alabama, Georgia, Louisiana, metropolitan Detroit, Michigan, New Jersey, North Carolina, Ohio, South Carolina, Tennessee, Illinois, and Texas. Women newly diagnosed with EOC and identified by the region’s cancer registry were eligible to participate if they self-identified as African American or Black, were 20–79 years of age at diagnosis (captures over 80% of cases),^26^ and could complete an interview in English. The median and mean times from diagnosis to enrollment were approximately 6 and 7 months, respectively. Eligible participants had histologically confirmed EOC and were centrally reviewed by an expert study pathologist. Women were considered to have EOC if the cancer originated in the ovary, fallopian tube, or specific parts of the peritoneum, or the International Classification of Diseases for Oncology, 3rd Edition (ICD-O-3) site codes C569, C570, or C481-488, respectively.^27^ A full list of eligible histology codes can be found in Supplemental Table 1; https://links.lww.com/EE/A379. Women enrolled in AACES were more likely to be women who had survived at least until the time of contact. It has been shown that the survival patterns in AACES are representative of women who survive at least 10 months out from diagnosis.^22^

#### California Cancer Registry

The CCR^25^ is a statewide population-based cancer registry that participates in the National Cancer Institute’s SEER Program. Mandated by state law, the CCR collects information about all cancers diagnosed in California. From the CCR, we identified all women in California who were newly diagnosed with primary EOC (ICD-O-3 site code C569, histology codes presented in Supplemental Table 1; https://links.lww.com/EE/A379) from 2004 to 2016. This study is covered under the Greater Bay Area Cancer Registry protocol approved by the IRB of the University of California, San Francisco.

Women with noninvasive tumors, not-first primary cancers, diagnoses based on autopsy or death certificate, or nongeocodable addresses were excluded from the analysis. We further restricted our main analysis to non-Hispanic Black women (hereafter referred to as Black women) who survived at least 10 months after EOC diagnosis to align it more closely with the AACES population. The impact of this restriction was evaluated in sensitivity analyses that included all women regardless of survival time.

### Exposure assessment

We obtained ambient PM_2.5_ exposure data from the publicly available Socioeconomic Data and Application Center air quality data set for health-related applications.^28^ Socioeconomic Data and Application Center includes yearly ambient PM_2.5_ levels (in µg/m^3^) estimated at a 1 km spatial resolution using a well-validated ensemble-based prediction model for the contiguous United States (2000–2016). As described by Di et al^29^, three machine-learning algorithms, random forest, neural network, and gradient boosting, included a variety of predictor variables from satellite data, land use, meteorological variables, and chemical transport model simulations to predict PM_2.5_. The ensemble model then combined these PM_2.5_ predictions with a generalized additive model that allowed for the contribution of each machine-learning algorithm to vary by location.^29^ The ensemble model was trained on PM_2.5_ levels measured at 2156 US EPA monitors, validated with 10-fold cross-validation, and produced high-resolution annual PM_2.5_ predictions with a mean R^2^ of 0.89 between predicted and monitored PM_2.5_.^29^

Daily NO_2_^30^ concentrations from 2000 to 2016 were estimated at 1 km spatial resolution using a well-validated ensemble machine-learning model, which integrated multiple predictor variables and three machine-learning algorithms.^31^ The model for annual NO_2_ exposure estimates demonstrated good performance with a mean R^2^ of 0.84 between predicted and monitored NO_2_.

In both cohorts, each participant’s address at diagnosis was geocoded and, with their latitude and longitude coordinates, linked to the average air pollutant exposures in the year of diagnosis and the 4 previous years. We considered three exposure periods: the year of diagnosis, the average of the year of diagnosis and the 2 years prior (i.e., a 3-year average), and the average of the year of diagnosis and the 4 years prior (i.e., a 5-year average).

### Outcome assessment

In these analyses, the outcome of interest was defined as the time between diagnosis and death from any cause or the last date of follow-up, in years. Overall survival was selected due to incomplete cause of death data and because the vast majority of women diagnosed with ovarian cancer die from this disease,^32^ limiting the impact of this constraint. Within AACES, vital status was collected and updated periodically via the cancer registries, the National Death Index, and LexisNexis Accurint. The most recent study-wide vital status update for AACES was complete for deaths that occurred through October 2024, the censoring date for surviving participants.

For the CCR, vital status was routinely determined through linkages to two primary sources—California state mortality files and the National Death Index. Follow-up for deaths for the CCR cohort was considered complete through 31 December 2021, which was used as the censoring date.

### Assessment of potential confounders

AACES collected information on potential confounders of the relationship between air pollution and all-cause mortality from participants from a thorough survey completed over the phone. For the CCR cohort, these data were extracted directly from the registry research dataset. Because data within the CCR cohort were abstracted primarily from medical and hospitalization records, and the data within the AACES cohort were additionally captured via surveys about lifestyle behaviors, there was a limited set of overlapping variables from which to select covariates for parallel analyses. The following variables were available for both cohorts to be harmonized and considered for analysis: year and age at diagnosis, the Charlson Comorbidity Index (a validated measure of comorbidity burden based on weighted chronic conditions, categorized as 0, 1, 2, 3+),^33^ insurance type (none, private only, Medicare only or Medicare & private, Medicaid, Other), marital status (never married, married or domestic partner, separated/divorced, or widowed), Yost/Yang neighborhood socioeconomic status index at the census tract level (nSES; composite index derived from US Census data reflecting neighborhood-level education, household income, poverty, unemployment, blue collar occupation, home value, and rental cost; index score was categorized into statewide quintiles with Q1 as the lowest and Q5 as the highest nSES),^34,35^ SEER tumor stage classification (localized, regional, and distant),^36^ and tumor histotype (high-grade serous, low-grade serous, endometrioid, clear cell, mucinous, carcinosarcoma, and other epithelial).^37^

Smoking behavior was not captured for the CCR cohort and, therefore, was not included in the primary analysis. However, sensitivity analyses adjusting for smoking status (never, current, former) were conducted within the AACES cohort.

### Statistical analysis

NO_2_ values were standardized so that a one-unit increase is equivalent to an increase of 10 ppb. PM_2.5_ was not standardized beyond the unit measure of 1 μg/m^3^. Both exposures were included in the models as linear terms, as there was no evidence of nonlinearity, assessed using cubic splines and Martingale residuals.

We used Weibull accelerated failure time models to assess the associations of exposure to PM_2.5_ or NO_2_ with survival in women with EOC and generated event time ratios (ETRs) and 95% confidence intervals (CIs). Weibull accelerated failure time models are well-suited for situations when the proportional hazards assumption is violated, and they offer an intuitive interpretation of effect estimates, reflecting changes in survival time driven by the covariates.^38^ The effect estimate represents a multiplicative effect on survival time, where an ETR <1 represents a shortened time to death, and an ETR >1 represents a longer time to death.

To account for the heterogeneity in survival time among individuals due to unmeasured covariates, we fit the models with a frailty term at the participant level.^39^ A frailty term represents a random effect that scales the survival time for each participant, capturing differences in the time to event not explained by the included covariates and reducing bias arising from unobserved heterogeneity.^40–42^

Three nested models with increasing numbers of covariates were fit. Model 1 was adjusted for the calendar year and age at diagnosis. Model 2 was further adjusted for the Charlson Comorbidity Index, insurance type, marital status, and nSES. Model 3 was further adjusted for the clinical covariates, such as stage and histotype. Model fit was assessed via the Akaike Information Criterion.^43^ To recover missing data in covariates used for adjusted analyses and preserve sample size, we implemented multiple imputation by chained equations, generating 50 imputed datasets.^44^ We then performed the adjusted analyses separately on each dataset and pooled the results using Rubin’s rules to obtain the final estimates.^45^

Analyses were conducted using R version 4.4.1 (Austria)^46^ and Stata version 18 (StatCorp, USA).^47^

### Comparative analysis

To compare our findings with those from two previous studies^19,20^ (hereafter referred to as Vieira’s and Villanueva’s studies by the names of the first authors) that examined the associations between air pollution exposures and ovarian cancer survival in California using CCR data, we examined association in the CCR cohort without restrictions on race, ethnicity, or survival time but limited to individuals 18 years or older at date of diagnosis. Other changes to our analyses described above included (1) use of the Cox Proportional Hazards model for assessing the association between exposure to PM_2.5_ and NO_2_ and EOC-specific survival, (2) standardizing exposure so that unit change represents an increase by an interquartile range (IQR) as in Villanueva’s study,^19^ or an increase from the 5th to the 95th percentile as in Vieira’s study,^20^ (3) grouping NO_2_ exposure into three categories (<20, 20–30, and >30 ppb) as in Villanueva’s study,^19^ (4) restricting population to women diagnosed at stages IIIC or IV when comparing with Vieira’s study,^20^ and (5) using a similar set of covariates with corresponding categories and inclusion of the “Unknown” category in certain cases. Similar to our main analyses, we used the PM_2.5_ and NO_2_ measures at the year of diagnosis. The interpretation of hazard ratios (HRs) differs from that of ETRs used in the main analysis: an HR >1 indicates a higher hazard of death, whereas an ETR >1 indicates a longer survival time.

A few differences remained in this analysis in comparison to the two previous studies: (1) distinct data sources and rules for assigning data to the cohort were used for measures of air pollution, (2) included diagnosis years were 2004–2016 instead of 1996–2014 in Villanueva’s study^19^ or 1996–2006 in Vieira’s study,^20^ (3) lack of adjustment for the National Comprehensive Cancer Network’s guideline-adherence, (4) cancer stage was described following the American Joint Committee on Cancer (AJCC) instead of the International Federation of Gynecology and Obstetrics staging system, and (5) lack of spatial analysis as in Vieira’s study.^20^ We used the AJCC rather than the SEER staging system in this comparative analysis, as it is closer to the International Federation of Gynecology and Obstetrics. To assess whether differences in study years might account for variation in findings, we additionally assessed the interaction of exposure to air pollution with the year at diagnosis, categorizing the latter into three groups: 2004–2007, 2008–2012, and 2013–2016.

## Results

### Population characteristics

The study population of Black women diagnosed with EOC included 540 women from the AACES cohort and 766 women from the CCR cohort. There were notable differences in characteristics between the AACES and CCR cohorts (Table 1). The AACES cohort included women diagnosed between 2010 and 2015, while the CCR cohort covered diagnoses from 2004 to 2016. Compared to CCR, the AACES cohort included a higher proportion of younger women (34% aged 50–59 vs. 27%) and no women aged 80 or older (0% vs. 8%). The AACES cohort also had more women diagnosed with localized-stage disease (23% vs. 15%) and higher proportions of high-grade serous (68% vs. 57%) and endometrioid histotypes (10% vs. 7%). In contrast, the CCR cohort had a higher percentage of tumors classified with “other” epithelial histotype (22% vs. 7% in AACES). A more specific classification of tumors in AACES is likely due to the central pathology review of all tumor records in the cohort.

**Table 1.** Characteristics of Black women diagnosed with epithelial ovarian cancer and survived ≥10 months postdiagnosis, AACES (2005–2010) and CCR (2004–2016) cohorts

	AACES n (%)	CCR n (%)
Total	540 (100)	766 (100)
Year of diagnosis		
2004–2006	-	168 (22)
2007–2009	-	185 (24)
2010–2012	245 (45)	168 (22)
2013–2015	295 (55)	182 (24)
2016	-	63 (8)
Age at diagnosis, years		
<50	113 (21)	185 (24)
50–59	186 (34)	205 (27)
60–69	154 (29)	193 (25)
70–79	87 (16)	124 (16)
80+	-	59 (8)
SEER stage		
Localized	125 (23)	114 (15)
Regional	50 (9)	133 (17)
Distant	336 (62)	492 (64)
Unknown	29 (5)	27 (4)
Histotype		
High-grade serous	368 (68)	436 (57)
Carcinosarcoma	16 (3)	20 (3)
Clear cell	21 (4)	28 (4)
Endometrioid	56 (10)	53 (7)
Low-grade serous	16 (3)	18 (2)
Mucinous	25 (5)	41 (5)
Other epithelial	38 (7)	170 (22)
Charlson comorbidity index		
0	229 (42)	385 (50)
1	125 (23)	166 (22)
2	71 (13)	67 (9)
3+	106 (20)	70 (9)
Unknown	9 (2)	78 (10)
Marital status		
Never married	126 (23)	278 (36)
Married/domestic partner	176 (33)	220 (29)
Separated/divorced/widowed	238 (44)	234 (31)
Unknown	-	34 (4)
Insurance type		
No insurance	49 (9)	18 (2)
Private only	177 (33)	364 (48)
Medicare only or Medicare + Private	135 (25)	161 (21)
Any Medicaid	111 (21)	176 (23)
Other	29 (5)	32 (4)
Unknown	39 (7)	15 (2)
Yost neighborhood SES, census tract level, state-based quintiles		
Q1 – Lowest	188 (35)	229 (30)
Q2	113 (21)	182 (24)
Q3	91 (17)	130 (17)
Q4	78 (14)	127 (17)
Q5 – Highest	53 (10)	79 (10)
Unknown	17 (3)	19 (2)
Smoking status		
Never	299 (55)	-
Former	186 (35)	-
Current	55 (10)	-

The AACES cohort included a higher proportion of women with two or more comorbidities (33% vs. 18% in CCR) and of women who were married or separated rather than single (77% vs. 60%). Insurance coverage patterns differed as well, with more women in AACES lacking insurance (9% vs. 2%) or having nonprivate insurance (51% vs. 48%). Neighborhood SES in CCR was somewhat more skewed toward higher quintiles compared with AACES, with a greater proportion in Q4 (17% vs. 14%) and a smaller proportion in Q1 (30% vs. 35%).

The proportion of missing data imputed using previously described methods varied by population characteristic, ranging from 2% for nSES (AACES) to 5% for stage (CCR, Table 1).

### Distribution of air pollutants

**Figure 1.**
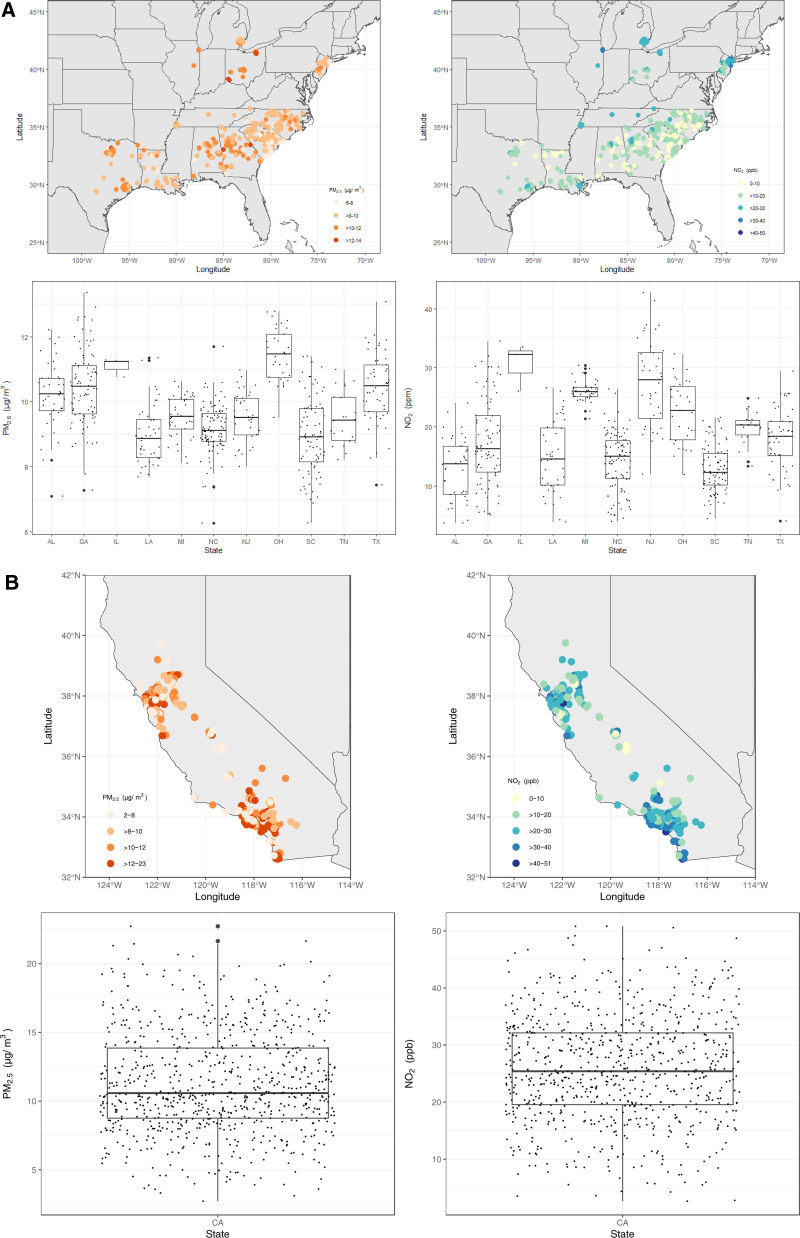
Distribution of assigned individual-level environmental concentrations of PM_2.5_ and NO_2_ in the study population, by cohort and state, AACES (2010–2015) and CCR (2004–2016) cohorts. AL, Alabama, CA, California, GA, Georgia, IL, Illinois, LA, Louisiana, MI, Michigan, NC, North Carolina, NJ, New Jersey, OH, Ohio, SC, South Carolina, TN, Tennessee, TX, Texas.

Environmental concentrations of both PM_2.5_ and NO_2_ were both higher for women in the CCR cohort compared to women in the AACES cohort (Table 2). The 5-year average PM_2.5_ exposures for women in CCR and AACES were 12.27 μg/m^3^ (standard deviation [SD] = 4.04) and 10.44 μg/m^3^ (SD = 1.12), respectively. Among the AACES sites, women from Illinois and Alabama had the highest 5-year average of PM_2.5_ exposures, 11.88 μg/m^3^ (SD = 0.62) and 11.10 μg/m^3^ (SD = 0.94), respectively. Conversely, women from Louisiana and South Carolina had the lowest 5-year average of PM_2.5_ exposures, 9.64 μg/m^3^ (SD = 0.85) and 9.78 μg/m^3^ (SD = 1.06), respectively. The 5-year average NO_2_ exposures for women in CCR and AACES were 28.11 ppb (SD = 9.97) and 18.30 ppb (SD = 7.21), respectively. Within AACES, women in the Chicago metropolitan area and New Jersey had the highest 5-year average of NO_2_ exposures, 31.86 ppb (SD = 6.26) and 29.24 ppb (SD = 7.00), respectively, and women from Alabama and South Carolina had the lowest exposures, 13.14 ppb (SD = 4.29) and 13.20 ppb (SD = 3.96), respectively. The distribution of air pollutants within each cohort and state can be found in Figure 1A and B, with participant latitudes and longitudes randomly jittered to anonymize and protect privacy.

**Table 2.** Distribution of assigned individual-level environmental concentrations of PM_2.5_ and NO_2_ in the study population, by cohort and state, AACES (2005–2010) and CCR (2004–2016) cohorts

Cohort	AACES												CCR
State (n)	Overall (540)	AL (40)	TX (53)	GA (86)	IL (3)	LA (44)	MI (40)	NC (102)	NJ (39)	OH (31)	SC (85)	TN (17)	CA (766)
	Mean (SD)												
PM_2.5_ (μg/m^3^)													
Year of diagnosis	9.73 (1.20)	10.16 (1.03)	10.43 (1.08)	10.48 (1.15)	11.09 (0.29)	8.98 (0.89)	9.54 (0.63)	9.17 (0.74)	9.54 (0.75)	11.39 (0.80)	8.94 (1.17)	9.64 (0.85)	11.29 (3.67)
3-year average	10.06 (1.14)	10.61 (1.04)	10.72 (0.91)	10.86 (1.04)	11.48 (0.78)	9.36 (0.91)	9.67 (0.41)	9.45 (0.60)	9.67 (0.50)	11.86 (0.61)	9.30 (1.03)	10.28 (1.00)	11.76 (3.78)
5-year average	10.44 (1.12)	11.10 (0.94)	10.87 (0.95)	11.29 (0.91)	11.88 (0.62)	9.64 (0.85)	10.04 (0.61)	9.82 (0.68)	10.02 (0.64)	12.12 (0.58)	9.78 (1.06)	10.86 (0.58)	12.27 (4.04)
NO_2_ (ppb)													
Year of diagnosis	17.52 (7.15)	13.13 (5.29)	17.99 (5.10)	17.45 (7.34)	30.59 (3.96)	15.00 (5.89)	26.17 (1.78)	14.49 (4.85)	27.82 (7.22)	22.15 (5.17)	12.86 (3.99)	20.16 (2.32)	25.81 (9.25)
3-year average	17.92 (7.10)	12.99 (4.31)	18.71 (5.21)	18.00 (7.11)	31.47 (5.48)	15.30 (5.83)	25.79 (1.16)	15.09 (4.98)	28.85 (6.77)	22.84 (5.41)	12.96 (3.75)	20.36 (2.52)	27.04 (9.58)
5-year average	18.30 (7.21)	13.14 (4.29)	19.28 (5.31)	18.24 (7.05)	31.86 (6.26)	15.62 (5.82)	26.12 (0.90)	15.57 (5.30)	29.24 (7.00)	23.64 (5.28)	13.20 (3.96)	20.98 (2.72)	28.11 (9.97)

AL, Alabama, CA, California, GA, Georgia, IL, Illinois, LA, Louisiana, MI, Michigan, NC, North Carolina, NJ, New Jersey, OH, Ohio, SC, South Carolina, TN, Tennessee, TX, Texas.

PM_2.5_ concentrations averaged over 1, 3, or 5 years were highly correlated with each other, as were those for NO_2_, in both the AACES and CCR cohorts, with Pearson’s *r* ranging from 0.83 to 0.99 (Supplemental Figure 1A and B; https://links.lww.com/EE/A379). The correlations between PM_2.5_ and NO_2_ were also strong in the CCR cohort (*r* ranging from 0.57 to 0.66), but substantially less so in the AACES cohort (*r* ranging from 0.23 to 0.27).

### Association between ambient air pollution and survival

The associations for PM_2.5_ or NO_2_ air pollutants with overall mortality were largely null in both the CCR and AACES cohorts, regardless of how many years of exposure were included in these measures, or which covariates were included in the analyses (Table 3).

**Table 3.** Event time ratios^a^ and 95% CIs for all-cause mortality in Black women with epithelial ovarian cancer who survived ≥ 10 months postdiagnosis in relation to air pollution exposure (PM_2.5_ and NO_2_), AACES (2005–2010) and CCR (2004–2016) cohorts

	Model 1 (Minimally adjusted)^b^		Model 2 (Fully adjusted)^c^		Model 3 (+ stage and histotype)^d^	
	AACES (n = 540)	CCR (n = 766)	AACES (n = 540)	CCR (n = 766)	AACES (n = 540)	CCR (n = 766)
PM_2.5_ (per 1 μg/m^3^)						
Year of diagnosis	0.99 (0.93, 1.06)	1.09 (0.97, 1.21)	0.99 (0.93, 1.06)	1.11 (0.99, 1.23)	1.00 (0.93, 1.07)	1.08 (0.97, 1.20)
3-Year average	1.01 (0.93, 1.08)	1.08 (0.97, 1.20)	1.02 (0.95, 1.10)	1.11 (0.99, 1.22)	1.01 (0.94, 1.10)	1.08 (0.97, 1.20)
5-Year average	0.99 (0.92, 1.07)	1.07 (0.95, 1.20)	1.00 (0.93, 1.08)	1.10 (0.97, 1.22)	1.01 (0.93, 1.10)	1.07 (0.95, 1.19)
NO_2_ (per 10 ppb)						
Year of diagnosis	0.97 (0.85, 1.09)	1.03 (0.96, 1.11)	1.00 (0.88, 1.12)	1.08 (0.99, 1.16)	1.06 (0.92, 1.21)	1.07 (0.99, 1.15)
3-Year average	0.97 (0.86, 1.09)	1.00 (1.00, 1.01)	1.00 (0.89, 1.12)	1.01 (1.00, 1.02)	1.05 (0.92, 1.20)	1.01 (1.00, 1.02)
5-Year average	0.94 (0.84, 1.06)	1.00 (1.00, 1.01)	0.97 (0.87, 1.09)	1.01 (1.00, 1.01)	1.02 (0.90, 1.16)	1.01 (1.00, 1.01)

a Event time ratios reflect a change in survival time associated with 1 μg/m^2^ increase in PM_2.5_ or a 10-ppb increase in NO_2,_ where estimates >1 represent increase in survival time and estimates <1 represent decrease in survival time.

b Adjusted for year and age at diagnosis.

c Adjusted for year, age at diagnosis, Charlson comorbidity index, insurance coverage, marital status, and nSES; multiple imputation used for missing covariate data.

d Adjusted for stage at diagnosis and histotype in addition to covariates from Model 2; multiple imputation used for missing covariate data.

In the minimally adjusted model for the AACES cohort, overall time to death among Black women with EOC was not associated with a 1-μg/m^3^ increase in PM_2.5_ concentration at the year of diagnosis (ETR = 0.99, 95% CI = 0.93, 1.06) or in any of the time periods before diagnosis (ETR 3-year average PM_2.5_ = 1.01, 95% CI = 0.93, 1.08; ETR 5-year average PM_2.5_ = 0.99, 95% CI 0.92, 1.07). Adjustment for additional covariates did not change this null association. In the minimally adjusted model for the CCR cohort of Black women with EOC, we observed a slight increase in survival time associated with a 1-μg/m^3^ increase in PM_2.5_ concentration, albeit with considerable uncertainty (ETR for PM_2.5_ measured at the year of diagnosis = 1.09, 95% CI = 0.97, 1.21; ETR 3-year average PM_2.5_ = 1.08, 95% CI = 0.97, 1.20; ETR 5-year average PM_2.5_ = 1.07, 95% CI = 0.95–1.20). Adjustment for additional covariates did not change this association.

Similarly, in the minimally adjusted model for the AACES cohort, overall time to death among Black women with EOC was not associated with a 10-ppb increase in NO_2_ concentration regardless of time window contributing to exposure estimates (year of diagnosis ETR = 0.97, 95% CI = 0.85, 1.09; 3-year average ETR = 0.97, 95% CI = 0.86, 1.89; 5-year average ETR = 0.94, 95% CI = 0.84, 1.06). Adjustment for other covariates did not change the result. In the CCR cohort, the findings were similar, where, in the minimally adjusted models, the ETRs for a 10-ppb increase in NO_2_ concentration were 1.03, 95% CI = 0.96, 1.11, 1.00, 95% CI = 1.00, 1.01, and 1.00, 95% CI = 1.00, 1.01, with PM_2.5_ measured at the year of diagnosis, the 3- and the 5-year average before diagnosis, respectively. Adjustment for additional covariates did not change this association.

Restricting survival time to ≥10 months did not affect the results, as estimates remained similar in a sensitivity analysis of the CCR cohort without survival time restrictions, suggesting that selection bias is unlikely to explain the results (Supplemental Table 2; https://links.lww.com/EE/A379). Furthermore, adjusting for smoking status in the AACES models did not change the results, suggesting that smoking was not a strong confounder in this context (Supplemental Table 3; https://links.lww.com/EE/A379).

### Comparative analysis

We expanded the CCR cohort to include all racial and ethnic groups and removed survival time restrictions, along with other adjustments outlined in Section *Comparative analysis* of *Materials and methods* to examine the comparability of findings with two previous CCR-based studies.^19,20^ Most population characteristics in this comparative analysis were similar to those reported in Villanueva’s study, including the distribution of age at diagnosis, race/ethnicity, nSES, marital status, tumor size, and tumor grade. However, several differences were observed: this study covered a different time period (2004–2016 vs. 1996–2014 in Villanueva), had a longer median survival time (48.8 vs. 34.5 months), a higher proportion of women enrolled in Medicaid (16 vs. 9%), a greater proportion with serous histotype (51 vs. 43%), and more women without comorbidities (61 vs. 48%, Supplemental Table 4; https://links.lww.com/EE/A379).

In this extended CCR cohort, the mean level of PM_2.5_ exposure at the time of diagnosis was 11.0 μg/m^3^ (SD = 3.7), and NO_2_ exposure was 25.7 ppb (SD = 9.6, see Supplemental Table 5; https://links.lww.com/EE/A379). Exposure levels varied by racial and ethnic group, with non-Hispanic Black women exposed to the highest PM_2.5_ concentrations (mean = 11.4 μg/m³, SD = 3.6) and among the highest NO_2_ concentrations (mean = 25.9 ppb, SD = 9.3), comparable to non-Hispanic White women (mean = 26.5 ppb, SD = 9.6, Supplemental Figure 2; https://links.lww.com/EE/A379).

In our extended study population, average PM_2.5_ exposure levels were lower than those reported by Villanueva (11.0 vs. 12.2 μg/m³), while average NO_2_ exposure levels were significantly higher (25.7 vs. 16.1 ppb; see Supplemental Table 5; https://links.lww.com/EE/A379). The IQR for PM_2.5_ was similar (4.4 and 4.7), enabling direct comparisons of estimates between the two studies.

In the adjusted analysis of the extended CCR cohort matching the methods of the two previous CCR-based studies^19,20^ (Table 4), we found no association between the hazard of dying from ovarian cancer and a 4.7 µg/m^3^ increase in PM_2.5_ concentration at the year of diagnosis (HR = 1.00, 95% CI = 0.97, 1.03). However, a weak association was observed among women diagnosed with stage IIIC or IV EOC, such that an increase in PM_2.5_ exposure from the 5th to the 95th percentile was associated with a 1.08-times higher hazard of dying from ovarian cancer (95% CI = 1.00, 1.16). No statistically significant interaction with time (i.e., year of diagnosis) was observed (Supplemental Table 6; https://links.lww.com/EE/A379).

**Table 4.** Hazard ratios^a^ and 95% CIs for EOC-specific mortality in women with epithelial ovarian cancer of all racial and ethnic groups in relation to air pollution exposure (PM_2.5_ and NO_2_), CCR cohort, in comparison with the data from previous CCR-based studies

	Estimates from this study (2004–2016)	Original estimates from Vieira et al^20^ (1996–2006)	Original estimates from Villanueva et al^19^ (1996–2014)
Cancer stages IIIC or IV			
* *PM_2.5_ (increase from 5th to 95th percentile)			
* *n	12,083	11,765	-
* * Unadjusted	1.08 (1.00, 1.16)	-	-
* * Adjusted^b^	1.08 (1.00, 1.16)	1.10 (1.01, 1.19)	-
All cancer stages			
* *PM_2.5_ (per IQR of 4.7 μg/m^3^)			
n	20,303	-	25,976
* * Unadjusted	1.03 (1.01, 1.06)	-	1.47 (1.43, 1.50)
* * Adjusted^c^	1.00 (0.97, 1.03)	-	1.44 (1.40, 1.47)
* *NO_2_ (categories)			
* * n	20,303	-	29,841
* * Unadjusted			
* * <20 ppb	Reference	-	reference
* * 20–30 ppb	1.03 (0.98, 1.08)	-	1.20 (1.16, 1.25)
* * >30 ppb	1.05 (1.00, 1.11)	-	3.03 (2.85, 3.22)
* * Adjusted^c^			
* * <20 ppb	Reference	-	reference
* *20–30 ppb	0.99 (0.94, 1.04)	-	1.30 (1.25, 1.36)
* *>30 ppb	0.97 (0.92, 1.03)	-	2.48 (2.32, 2.66)

a Hazard ratios reflect differences in the hazard of dying from ovarian cancer, where estimates >1 represent increase in hazard of dying and estimates <1 represent decrease in hazard of dying.

b Adjusted for age at diagnosis, insurance coverage, nSES, race and ethnicity, stage, histology, tumor grade. Vieira et al^20^ additionally adjusted for the National Comprehensive Cancer Network’s guideline adherence.

c Adjusted for age at diagnosis, insurance coverage, nSES, race and ethnicity, stage, histology, tumor grade, year at diagnosis, marital status, Charlson comorbidity index, and tumor size. Villanueva et al^19^ additionally adjusted for the National Comprehensive Cancer Network’s guideline adherence.

In a similar adjusted analysis for the NO_2_ exposure, we found little evidence of association with EOC survival (HR for NO_2_ 20–30 ppb: 0.99, 95% CI = 0.94, 1.04; HR for NO_2_ >30 ppb: 0.97, 95% CI = 0.92, 1.03) among women of all racial and ethnic groups.

These results are only partially consistent with the two previous studies conducted with CCR data (Table 4).^19,20^ The HR of 1.08 (95% CI = 1.00, 1.16), associated with an increase from the 5th to the 95th percentile of PM_2.5_ concentrations, was comparable to the 1.10 (95% CI = 1.01, 1.19) reported in Vieira’s study.^20^ In contrast to Villanueva’s study,^19^ which reported a strong association between exposure to both PM_2.5_ and NO_2_ and ovarian cancer survival, we did not observe a similar result.

## Discussion

We leveraged two large datasets of Black ovarian cancer survivors, applying high-resolution air pollution exposure estimates derived from well-validated ensemble-based prediction models to assess the associations between exposure to PM_2.5_ and NO_2_ and survival. We found no association between outdoor PM_2.5_ and NO_2_ pollution and overall survival among Black women diagnosed with EOC across diverse US regions, with exposure assessed at the residential address and recorded at diagnosis year or averaged over the 3- or 5-year period leading up to and including the year of diagnosis. Regardless of the data source (AACES or CCR), the same results were observed. This lack of observed association was robust to changing covariates or extending the analysis to other racial and ethnic groups.

This study contributes to the limited body of research examining the relationship between ambient air pollution and ovarian cancer survival. Existing evidence remains mixed: while some studies have reported positive associations of ovarian cancer mortality with specific pollutants such as NO_2_ and PM_2.5,_^17–20,48^ others, including ours, have found no such link.^21,22^ Apart from the fact that most of these studies are based on data from China and Taiwan, where air pollution levels are higher than in the United States, variations in findings may also stem from differences in air pollution exposure estimation methods, timing of exposure assessment, statistical modeling approaches, and study population characteristics. Our study stands out using the advanced high-resolution air pollution exposure estimates, a larger geographic scope, and a focus on the Black women population. To explore the differences in findings further, we conducted a comparative analysis with two positive studies that examined a similar underlying population from the CCR but included all racial and ethnic groups.^19,20^

Similar to our primary analysis of Black women with EOC, our comparative analysis, which included all racial and ethnic groups, found little evidence of association between PM_2.5_ or NO_2_ exposure and survival. These findings contrast with previous CCR-based studies, despite our efforts to closely replicate their modeling strategies, covariate selections, and population characteristics. To the extent of the point estimate, our results aligned with Vieira’s study,^20^ which reported an increased hazard of ovarian cancer mortality with rising PM_2.5_ concentrations from the 5th to the 95th percentile in women with stage IIIC and IV cancer (HR 1.08, 95% CI = 1.00, 1.16, in our analysis vs. 1.10, 95% CI = 1.01, 1.19, in Vieira’s study). In contrast, our results did not replicate the strong associations reported in Villanueva’s study, which found significantly elevated risks for PM_2.5_ (IQR HR = 1.44, 95% CI = 1.40, 1.47) and NO_2_ (>30 ppb vs. <20 ppb: HR = 2.48, 95% CI = 2.32, 2.66).^19^ Our findings highlight that even after accounting for differences in study population characteristics and statistical modeling approaches, results can still vary, likely due to differences in how air pollution exposure is estimated. For example, Villanueva study relied on raw monitoring data, whereas our study used high-resolution modeled estimates that incorporate multiple predictors. Another important difference is that in Villanueva’s study, exposure was averaged over each individual’s survival time, which may have biased estimates upward, as longer-surviving individuals were more likely to have lower average exposures due to declining pollution levels.

Our primary analysis focused on Black women, a historically disadvantaged group that often resides in neighborhoods affected by systemic disinvestment, resulting in poor social, built, and physical environments, including poorer air quality.^13,49^ While limited, prior research has also provided some insight into this issue. Villanueva’s study provided stratified estimates for the association between PM_2.5_ and NO_2_ exposure and ovarian cancer survival across different racial and ethnic groups.^19^ Notably, among all racial and ethnic groups, non-Hispanic Black women had the lowest HR for ovarian cancer mortality in relation to increased PM2.5 exposure (IQR HR = 1.21, 95% CI = 1.07, 1.37; n = 1,238). A similar trend was observed for NO_2_ exposure with even higher uncertainty in the estimate (HR for 20–30 ppb vs. <20 ppb = 1.14, 95% CI = 0.95, 1.37; n = 1,416). These findings may partly explain why we did not observe a strong association between PM_2.5_ or NO_2_ exposure and survival of Black women with ovarian cancer in our study. Consistent with this, in our extended CCR analysis, Black women experienced higher average air pollution levels with a narrower distribution than other racial and ethnic groups, which may have weakened the association between air pollution exposure and survival in this group. Alternatively, other factors, such as comorbidities, treatment, and healthcare access characteristics, may be more strongly associated with survival among Black women than air pollution, relative to other racial and ethnic groups.

Although the epidemiological evidence to date, including findings from our study, remains inconclusive regarding the relationship between air pollution and ovarian cancer outcomes, several biological mechanisms have been proposed to explain this potential link. Prolonged exposure to air pollution is hypothesized to contribute to inflammation and oxidative stress processes.^50,51^ This hypothesis is supported by epidemiological studies investigating the interaction between air pollution exposure and factors such as antioxidant-rich diets or oxidative balance scores in the context of ovarian cancer survival. These studies found that the adverse effects of air pollution were mitigated by healthier diets or higher oxidative balance scores.^52,53^ Another epidemiologic study leveraging data from a large nationwide cohort confirmed a linear positive association between levels of NO_2_ exposure and C-reactive protein levels in women.^54^ Given the biological plausibility of air pollution influencing ovarian cancer outcomes, further research is needed to refine exposure assessment—such as incorporating time-varying long-term exposure—and to account for population-specific factors, including potential effect modification by occupation, physical activity, and neighborhood stressors.

### Limitations

Because the CCR relied on registry data, behavioral factors such as smoking status or clinical factors like body mass index were incomplete and could not be selected for inclusion in this parallel analysis. While these were collected in surveys conducted in AACES, including these in the models for one cohort only would result in a lack of comparability between models.

Another limitation of our study is the potential misclassification of exposure. We measured the exposures to air pollutants at one point in time and were unable to account for factors such as time spent indoors, time spent in traffic, historical residential addresses, or residential relocation during the follow-up period, thus not reflecting the time-varying air pollution exposure. Notably, one study found no statistically significant association between ovarian cancer incidence and time-varying PM_2.5_ exposure but reported a significant relationship with time-varying NO_2_ exposure.^16^ Future research should leverage prospective cohort data to better assess how everyday and residential mobility influence the measurement of air pollution exposure.

### Strengths

This investigation is noteworthy for its robust methodology. It assesses diverse geographical regions over a wide span of years. Women in this study come from 12 different states across the United States, including states in the Northeast, Midwest, Southeast, Southwest, & the West, and the range of their diagnoses spans over a decade. This approach allowed us to capture a substantial variation in air pollution across rural and metropolitan areas nationwide, as well as changes in particulate matter levels over time due to declining emissions, which occurred at different rates across sociodemographic groups.^55^

In addition, having the same results from parallel analyses from studies with different study designs provides stronger evidence for the observed lack of association in this population. For example, because AACES utilized rapid case ascertainment in enrollment and collected additional survey data, factors of familiarity and comfort with the research apparatus likely influenced who actively participated in the study. In contrast, the CCR cohort relies on data that did not require direct participant engagement, thereby including women who might have been excluded due to differential response, loss to follow-up, or survival bias. Nevertheless, the results were similar despite these differences.

Our approach for estimating air pollution exposure offers several strengths over previous approaches. Unlike the two previous studies that relied on sparse monitoring station data linked to census tracts,^19,20^ we leveraged high-resolution predictions of PM_2.5_ and NO_2_ concentrations in 1 km grid cells. This fine-scale resolution reduces measurement error and enhances the accuracy of exposure estimates. Our exposure estimates are based on predictions from three advanced machine-learning models—neural network, random forest, and gradient boosting—and include satellite data, meteorological variables, land-use variables, elevation, and chemical transport model predictions.^29–31^ This comprehensive and sophisticated modeling framework ensures robust and precise air pollution estimates, thereby improving the reliability of our findings on the impact of air pollution on ovarian cancer survival. Moreover, we measured exposure up to 4 years prediagnosis—the longest window our data allowed. Although evidence for a latency period is limited for air pollution and ovarian cancer survival,^18,53^ long-term exposure is generally considered more impactful than short-term exposure.

Further, this is the first study examining the association between air pollution and ovarian cancer survival in the US regions other than California. It is also the first study of this kind to focus on Black women, a group historically vulnerable to social and health inequities and experiencing higher mortality rates from EOC.^2^ The largest study in this area included many women with EOC, but less than 5% of the study population identified as non-Hispanic Black.^19^

Our statistical approach minimized bias in our study by using a parametric Weibull distribution to account for nonproportional baseline hazards, incorporating individual frailty to address residual heterogeneity in survival times, and applying multiple imputation to handle missing covariate data.

Additionally, this is the first study to attempt replication of previous analyses. This effort allowed us to highlight the sensitivity of findings regarding the relationship between air pollution and ovarian cancer survival to variations in data sources and methods for air pollution exposure estimation.

## Conclusion

In summary, this study found the lack of a significant association between outdoor air pollution exposure (PM_2.5_ and NO_2_) and ovarian cancer survival among Black women across diverse geographical regions of the United States. While outdoor air pollutants like PM_2.5_ and NO_2_ have been hypothesized to influence cancer outcomes through mechanisms such as inflammation and oxidative stress, our findings suggest that these exposures may play a marginal role in ovarian cancer survival. The consistency of null results across cohorts with varied data collection methodologies strengthens the validity of our findings, underscoring the importance of exploring other factors that may more substantially impact survival in this population, such as access to care, other neighborhood-level social and built environment factors,^56–58^ and comorbidities.^59^ This study also highlights the need for future research to refine air pollution exposure assessments and investigate potential interactions with social determinants of health, particularly for historically marginalized populations. By centering the experience of Black women disproportionately affected by health inequities, this study contributes to ongoing efforts to better understand and address disparities in ovarian cancer outcomes.

## Conflicts of interest statement

The authors declare that they have no conflicts of interest with regard to the content of this report.

## Acknowledgments

We would like to acknowledge the AACES interviewers Brandy Arredondo, Rachel Boehm, Dannelle Charles, Melody Chavez, Lauren Dempsey, Kierstin Faw, Juliana Fucinari, Mary Kan, Mary Beth Kolbicz, Myneka Macenat, Arianna Mason, Juana Paniagua, Maelia Pendley, and Olga Aranzabal. We also acknowledge the individuals responsible for facilitating case ascertainment across the sites, including Kevin Ward and Mackenzie Crawford (Georgia); Tingting Li and Lauren Maniscalco (Louisiana); Lisa Paddock and Wendy Cedeno (New Jersey); Paul Terry (Tennessee); Ann Hamilton (California), Mary Beth Kolbicz (Michigan); Cynthia Webb, JoElla Marting, and Heather Tipaldos (North Carolina); and Maxwell Akonde, Stephanie Chiodini, and Deb Hurley (South Carolina).

## Supplementary Material


