# Supplementary material for: Ambient air pollution and survival among Black women with epithelial ovarian cancer across diverse geographical regions of the United States
Source: Environ Epidemiol. 2025 Oct 17;9(6):e426. doi: 10.1097/EE9.0000000000000426 (PMC12537266; doi:10.1097/EE9.0000000000000426)
Supplement: Supplementary file 1 [file ee9-9-e426-s001.pdf]

Supplemental Table 1. ICD-O-3 Codes for Participants in AACES and CCR.

ICD-O-3 Code	AACES	CCR
8000	X	
8004	X	
8005	X	
8010	X	X
8011	X	
8020	X	
8021	X	X
8022	X	
8032	X	
8046	X	X
8050	X	X
8120	X	X
8140	X	X
8144	X	
8255	X	X
8260	X	X
8290	X	
8310	X	X
8313	X	X
8323	X	X
8380	X	X
8381	X	X
8382	X	
8383	X	
8410	X	
8440	X	X
8441	X	X
8442	X	X
8443	X	
8444	X	
8450	X	X
8460	X	X
8461	X	X
8462	X	
8470	X	X
8471	X	X
8472	X	
8474	X	
8480	X	X
8481	X	X

ICD-O-3 Code	AACES	CCR
8482	X	
8560	X	
8570	X	X
8575	X	
8950	X	X
8951	X	X
8980	X	X
9000	X	X
9111	X	
9014	X	
9015	X	X

Supplemental Figure 1. Correlation between air pollutant measures by cohort.

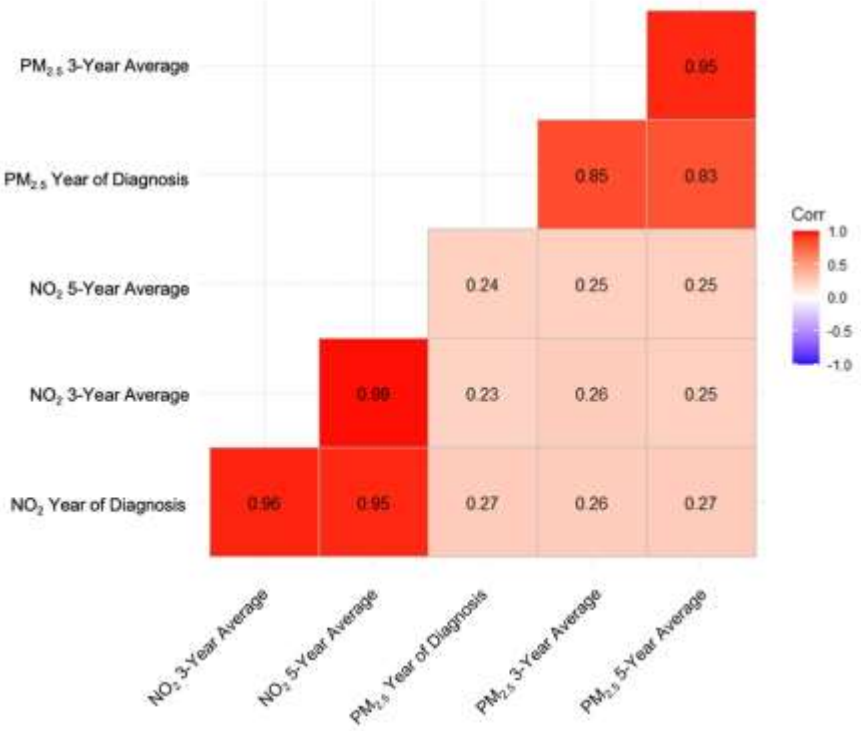

1A. AACES

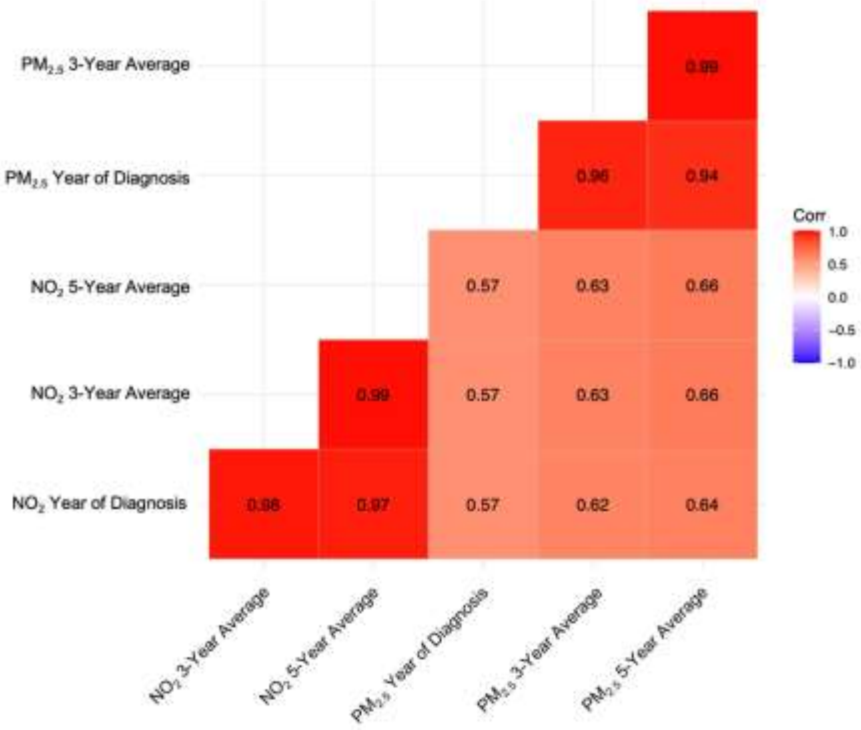

1B. CCR

Supplemental Table 2. Event time ratios[a] and 95% CIs for all-cause mortality in Black women with epithelial ovarian cancer in relation to air pollution exposure ($PM_{2.5}$ and NO_2), <u>without restriction on survival time</u>, CCR (2004-2016)

	**CCR (n=1,129)**		
	**Model 1 (Minimally adjusted)[b]**	**Model 2 (Fully adjusted)[c]**	**Model 3 (+ stage and histotype)[d]**
**$PM_{2.5}$ (per 1 μg/m^3)**			
Year of diagnosis	1.05 (0.84, 1.26)	1.00 (0.81, 1.19)	0.90 (0.75, 1.05)
3 Year average	1.07 (0.87, 1.27)	1.04 (0.85, 1.23)	0.93 (0.79, 1.08)
5 Year average	1.05 (0.84, 1.26)	1.02 (0.82, 1.22)	0.92 (0.76, 1.07)
**NO_2 (per 10 ppb)**			
Year of diagnosis	1.04 (0.90, 1.18)	1.03 (0.90, 1.17)	1.01 (0.88, 1.13)
3 Year average	1.00 (0.99, 1.02)	1.00 (0.99, 1.02)	1.00 (0.99, 1.01)
5 Year average	1.00 (0.99, 1.02)	1.00 (0.99, 1.02)	1.00 (0.99, 1.01)

Abbreviation: $PM_{2.5}$ – particulate matter of 2.5 micrometers or smaller in diameter, NO_2 – nitrogen dioxide, CI – confidence interval, *** – statistically significant at <0.001 level, ** – at <0.01 level, * – at <0.05 level.

[a]Event time ratios reflect change in survival time associated with 1 μg/m^2 increase in $PM_{2.5}$ or 10 ppb increase in NO_2, where estimates >1 represent increase in survival time and estimates <1 represent decrease in survival time.

[b]Adjusted for year and age at diagnosis.

[c]Adjusted for year, age at diagnosis, Charlson comorbidity index, insurance coverage, marital status, and nSES; multiple imputation used for missing covariate data.

[d]Adjusted for stage at diagnosis and histotype in addition to covariates from Model 2; multiple imputation used for missing covariate data.

Supplemental Table 3. Event time ratios[a] and 95% CIs for all-cause mortality in Black women with epithelial ovarian cancer who survived ≥ 10 months post-diagnosis in relation to air pollution exposure ($PM_{2.5}$ and NO_2), adjusted for smoking status, AACES (2005-2010)

	AACES (n=540)	
	**Smoking[b]**	**Smoking with Histotype & Stage[c]**
**$PM_{2.5}$ (per 1 μg/m^3)**		
Year of diagnosis	0.99 (0.93, 1.06)	1.00 (0.93, 1.08)
3 Year average	1.02 (0.95, 1.10)	1.01 (0.94, 1.10)
5 Year average	1.00 (0.93, 1.08)	1.01 (0.93, 1.10)
**NO_2 (per 10 ppb)**		
Year of diagnosis	0.99 (0.88, 1.12)	1.06 (0.93, 1.21)
3 Year average	1.00 (0.89, 1.12)	1.05 (0.92, 1.20)
5 Year average	0.97 (0.87, 1.09)	1.02 (0.90, 1.16)

Abbreviation: $PM_{2.5}$ – particulate matter of 2.5 micrometers or smaller in diameter, NO_2 – nitrogen dioxide, CI – confidence interval, *** – statistically significant at <0.001 level, ** – at <0.01 level, * – at <0.05 level.

[a]Event time ratios reflect change in survival time associated with 1 μg/m^2 increase in $PM_{2.5}$ or 10 ppb increase in NO_2, where estimates >1 represent increase in survival time and estimates <1 represent decrease in survival time.

[b]Adjusted for year, age at diagnosis, Charlson comorbidity index, insurance coverage, marital status, nSES, and smoking; multiple imputation used for missing covariate data.

[c]Adjusted for stage at diagnosis and histotype in addition to covariates from previous smoking model; multiple imputation used for missing covariate data

Supplemental Table 4. Characteristics of women of all racial and ethnic groups diagnosed with epithelial ovarian cancer, CCR cohort, in comparison with the data from Villanueva et al.[1]

	CCR, 2004-2016<br>n (%)	Villanueva *et al.*[1], 1996–2014<br>n (%)
**Total**	20,303 (100)	29,841 (100)
**Age at diagnosis, Median (SD)**	61 (14.2)	60 (14.9)
**Survival time (months), Median (SD)**	48.8 (53.4)	34.5 (56.4)
**Race/ethnicity**		
Asian/Pacific Islander	2,697 (13)	3,564 (12)
Hispanic	4,303 (21)	5,749 (19)
Non-Hispanic Black	969 (5)	1,416 (5)
Non-Hispanic White	12,189 (60)	18,917 (63)
Other	145 (1)	195 (1)
**Yost Neighborhood SES, census tract level, state-based quintiles**		
Q1 – Lowest	2,746 (14)	4,037 (14)
Q2	3,627 (18)	5,434 (18)
Q3	4,262 (21)	6,322 (21)
Q4	4,734 (23)	6,860 (23)
Q5 – Highest	4,934 (24)	7,188 (24)
**Insurance type**		
No insurance	493 (2)	889 (3)
Private only/Managed care	10,326 (51)	14,149 (47)
Medicare	5,266 (26)	7,652 (26)
Medicaid	3,294 (16)	2,725 (9)
Other	547 (3)	3,824 (13)
Unknown	377 (2)	602 (2)
**Marital status**		
Single	9,830 (48)	14,686 (49)
Married	10,473 (52)	15,155 (51)
**Stage**[a]		
I & II	6,330 (31)	9,733 (29)
III	7,708 (38)	11,262 (33)
IV	6,265 (31)	8,846 (38)
**Tumor size**		
<50	2,965 (15)	3,734 (13)
50-99	4,328 (21)	5,884 (20)
≥100	7,048 (35)	9,335 (31)
Unknown	5,962 (29)	10,888 (36)
**Tumor grade**		

1	1,541 (8)	2,374 (8)
2	2,416 (12)	4,359 (14)
3	6,496 (32)	10,050 (34)
4	3,865 (19)	4,191 (14)
Unknown	5,985 (29)	8,867 (30)
**Histology**		
Serous	10,339 (51)	12,854 (43)
Carcinoma, NOS	3,311 (16)	3,178 (11)
Clear cell	1,393 (7)	1,829 (6)
Endometrioid	2,276 (11)	3,318 (11)
Mucinous	1,270 (6)	1,900 (6)
Other	1,714 (9)	6,762 (23)
**Charlson comorbidity index**		
0	12,343 (61)	14,218 (48)
1	3,515 (17)	6,806 (23)
2+	2,629 (13)	6,725 (22)
Unknown	1,816 (9)	2,092 (7)

Abbreviation: CCR – California Cancer Registry, SD – standard deviation, SES – socioeconomic status, AJCC – The American Joint Committee on Cancer, NOS – not otherwise specified.
[a]The American Joint Committee on Cancer (AJCC) staging system was used in our analysis, and the International Federation of Gynecology and Obstetrics (FIGO)—in Villanueva et al.

Supplemental Table 5. Distribution of assigned individual-level environmental concentrations of $PM_{2.5}$ and NO_2 in women of all racial and ethnic groups, CCR cohort, in comparison with the data from Villanueva et al.[1]

	CCR, 2004-2016 (n=20,303)	Villanueva *et al.*[1], 1996–2014 (n=29,841)
**$PM_{2.5}$ (μg/m^3)**[a]		
Mean	11.0	12.2
SD	3.7	3.6
IQR	4.7	4.4
**NO_2 (ppb)**[a]		
Mean	25.7	16.1
SD	9.6	6.6
IQR	13.0	8.4

Abbrevation: CCR – California Cancer Registry, SD – standard deviation, IQR - interquartile range.
[a]In this analysis, PM2.5 and NO2 assigned concentrations reflect the average air pollutant exposures in the year of diagnosis. In Villanueva et al., pollution data was averaged over each woman's survival period.

Supplemental Figure 2. Distribution of assigned individual-level environmental concentrations of $PM_{2.5}$ and NO_2 by race and ethnicity, CCR, 2004-2016

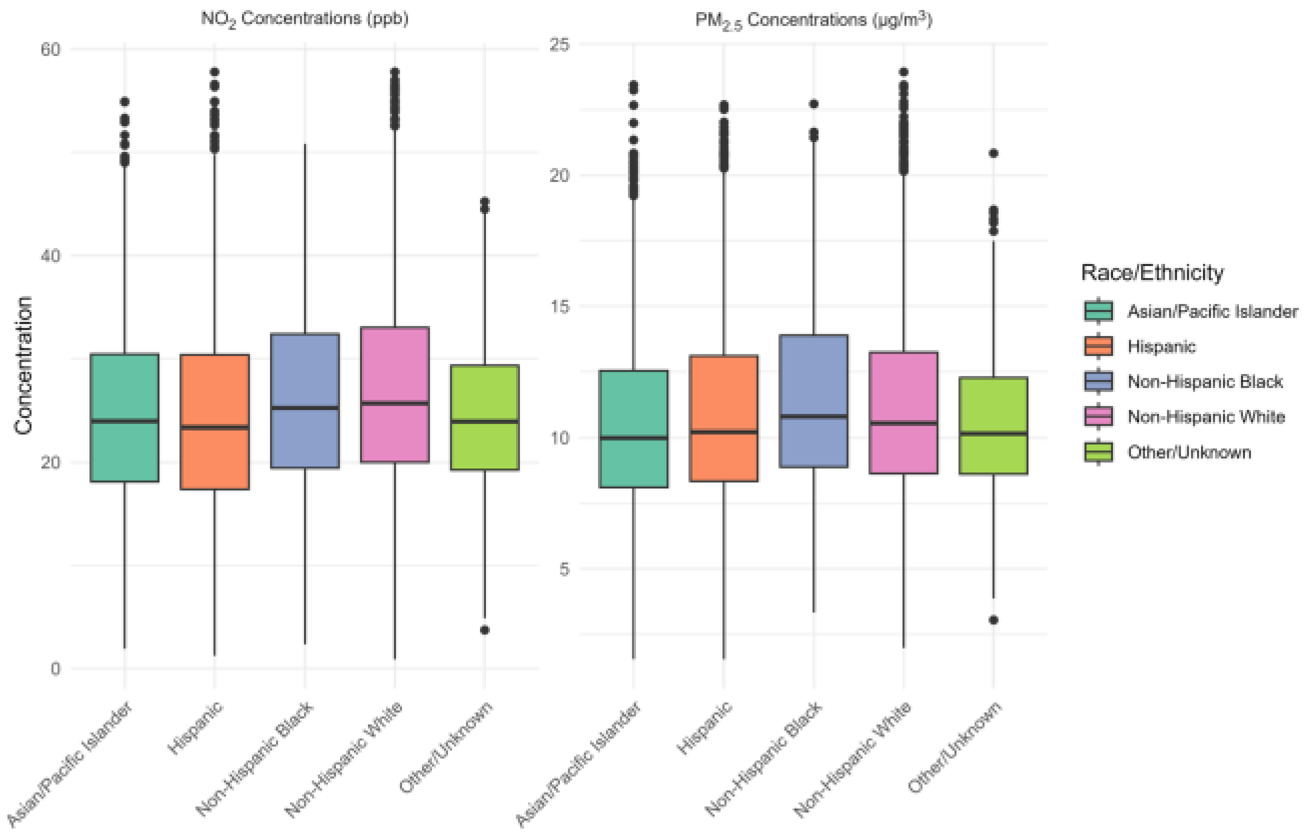

Supplemental Table 6. Global test of interaction between air pollution exposure ($PM_{2.5}$, NO_2) and year of diagnosis (2004-2007, 2008-2012, 2013-2016) on hazard ratios, CCR cohort

	P-value for the test of interaction
**Cancer stages IIIC or IV**	
$PM_{2.5}$ (increase from 5th to 95th percentile)[a]	0.210
**All cancer stages**	
$PM_{2.5}$ (per IQR of 4.7 μg/m^3)[b]	0.064
NO_2 (categories: < 20 ppb, 20-30 ppb, >30 ppb)[b]	0.111

Abbreviation: $PM_{2.5}$ – particulate matter of 2.5 micrometers or smaller in diameter, NO_2 - nitrogen dioxide.

[a]Adjusted for age at diagnosis, insurance coverage, nSES, race and ethnicity, stage, histology, tumor grade.

[b]Adjusted for age at diagnosis, insurance coverage, nSES, race and ethnicity, stage, histology, tumor grade, marital status, Charlson comorbidity index, and tumor size.

References:

1. Villanueva C, Chang J, Ziogas A, Bristow RE, Vieira VM. Ambient air pollution and ovarian cancer survival in California. *Gynecol Oncol*. 2021;163(1):155-161. doi:10.1016/j.ygyno.2021.07.036
